# Theory of brain complexity and marital behaviors: The application of complexity science and neuroscience to explain the complexities of marital behaviors

**DOI:** 10.3389/fnhum.2023.1050164

**Published:** 2023-03-07

**Authors:** Gholam Reza Nikrahan

**Affiliations:** ^1^Department of Psychology, Farhangian University, Tehran, Iran; ^2^Department of Psychology, University of Isfahan, Isfahan, Iran

**Keywords:** complex adaptive system, marital quality, marital relationship, marital stability, neuroscience, theories of marriage

## Abstract

The extant theories on the quality and stability of marital relationships have some difficulties in explaining some of the complexities of marital behaviors. The present article is an initial attempt to explain the complexities of marital behaviors based on the science of complexity and neuroscience. This article proposes a new theoretical framework relying on this simple argument that marital behaviors, as one of the most complex human behaviors, are the product of one’s brain’s complex adaptive system (CAS). Hence, to understand the complexities of marital behaviors, a movement toward familiarity with the brain’s CAS involved in marital behaviors needs to be started. The article presents the theory of brain complexity and marital behaviors (BCM) and outlines its assumptions, concepts, and propositions. Then, BCM is compared with the extant theories on happy and stable marriage, and finally, it was concluded by discussing the testability and the potential application of the theory. This article might inspire interdisciplinary studies of marital relationships, complex systems, and neuroscience and may have considerable practical implications.

*“The next century [21st] will be the century of complexity” – Stephen Hawking*

## 1. Introduction

The existing theories and models on the quality and stability of marital relationships have made unique and worthwhile contributions to the/an understanding of marriage. However, they have some difficulties in explaining some of the complexities of the marital relationship. For example, behavioral models – which focus on the quality of the couple’s interactions and communication skills – have ignored the underlying sources of spouses’ interaction. In addition, behavioral theories do not explain how couples with insufficient skills can improve on their own (Karney and Bradbury, 1995). Also, behavioral theories do not explain why some couples with negative interaction patterns continue their marriage despite long-term conflicts (Hawkins and Booth, 2005).

Similarly, social exchange theory of marriage (Levinger, 1965, 1976), explains why marriages are stable or unstable, considering the rewards, costs, alternatives, and barriers to leaving a relationship. However, how a marriage that was initially stable becomes unstable over time or vice versa is not in its scope (Karney and Bradbury, 1995). Furthermore, social exchange theory does not consider that two different people in identical situations may behave differently in low reward conditions. Clark and Mills (1979) emphasized that marriage is communal, not an exchange relationship. They believe that social exchange theory deduces human behaviors and communications as a purely rational process raised from economic theories. According to attachment theories of marriage (Hazan and Shaver, 1987, 1994), the quality and stability of adult romantic relationships depend on the attachment styles of partners developed in their infancy, in addition to the fulfillment of their basic needs for care, comfort, and sexual gratification. However, attachment theories do not explain why, under certain circumstances, people with insecure attachment styles can have stable relationships or why couples with the same attachment styles and the same levels of satisfaction with their needs may have different marriage destinations (Karney and Bradbury, 1995).

According to crisis theory (Hill’s 1949; McCubbin and Patterson, 1982) the level of the couple’s resources and their definitions of stressful events moderate the effects of these events and crises on the quality and stability of a relationship. However, as Karney and Bradbury (1995) pointed out, “rarely have crisis theorists addressed the specific coping responses that lead to either adaptation or maladaptation (Karney and Bradbury, 1995, p. 7).” Similarly, the vulnerability–stress–adaptation (VSA) model believes that adaptive processes and personal characteristics can interact with stressful events and enduring vulnerabilities and affect the couple’s level of adaptation to marital difficulties (Karney and Bradbury, 1995). However, the VSA model mentions “adaptive processes” but does not provide details about the content and sources of these processes. Additionally, the VSA model does not explain how these processes translate into positive behaviors that lead to a happy and stable marriage.

Furthermore, existing theories are facing some problems in the explanation of some complex phenomena such as non-linearity in dynamic changes in marital relationship, sudden changes in the marital relationship and its transformation into an entirely different relationship (phase transition), significant changes due to the small events (butterfly effect), the importance of starting values (positively or negatively) in marital interactions, the existence of attractors in marital interactions, difficulty in returning the relationship to its previous state after the occurrence of critical events such as a betrayal (hysteresis) (Gottman, 2014), and the possibility of self-repairing processes in marriages without external intervention (self-organization and adaptation) (Karney and Bradbury, 1995).

Marital relationship is very complex, and the brain has to take many variables into account when making decisions about the adaptive behaviors in a marital relationship. The present article raises the idea that untying the knot of complexities of the marital relationship is possible only when one accepts this simple argument that marital behavior, as one of the most complex human behaviors, is the product of their brain’s complex function. Therefore, to understand the complexities of marital behaviors, there is no choice but to become familiar with the brain’s complex system (CS), its subsystems, and how they interact to consider all of the variables and reach a final decision on marital behaviors; the fact that the existing theories have not taken it into account. Thus, this article aims to elucidate this idea by integrating evidence in the literature of marital relationships studies, CSs, neuroscience, and the author’ empirical work, which results in the “theory of brain complexity and marital behaviors” (BCM).

In the following sections, CSs’ features will be briefly discussed, followed by a discussion on the initial attempt of the author to discover the brain’s complex adaptive system (CAS) involved in marital behaviors. The BCM is then introduced, and its assumptions, concepts, and propositions are outlined in detail. BCM is compared with the extant theories, and ultimately, the conclusion is made by discussing the testability and the potential applications of the theory.

## 2. Complex systems, their key concepts, and features

A CS is a system that consists of numerous agents that interact with one another and potentially with their environment. These interactions lead to the emergence of a global property or collective behavior that is not observed by understanding each agent. Examples of CSs are the human brain, social and economic organizations, Earth’s global climate, and ant colonies.

**FIGURE 1:**
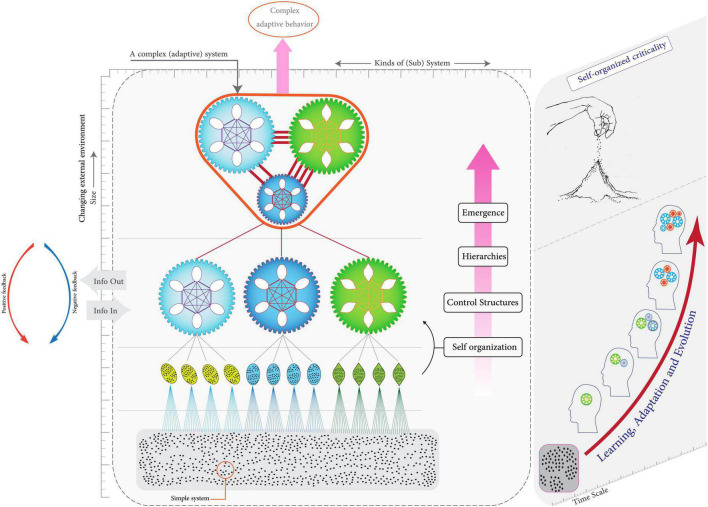
Characteristics of complex adaptive systems (CAS) and how they work. A CAS is a system that consists of many adaptive agents which interact with each other and their external environment. The interaction of components leads to collective behaviors which are different/greater than the sum of the parts (emergence). CAS evolves over time.

Complex adaptive systems are particular kinds of CSs that are able to learn from experience, change, and evolve. See Figure 1 for more details on the features of CASs. Although readers are familiar with some of these features in family systems theory, these features are reviewed and completed in order to understand the structure and function of the brain’s CAS involved in marital behaviors:

### 2.1. Numerosity

To generate CSs, more than a handful of individual agents have to be engaged in many interactions (Ladyman et al., 2013; Ladyman and Wiesner, 2020).

### 2.2. Emergence

The CS has different properties from those of the sum of its elements. In the ant colonies, the behavior of each ant is relatively simple compared to what the system does as a whole, such as building bridges and nests and raising aphid “livestock.” No single ant will know to undertake such collective tasks on its own. Just as a neuron does not know what commitment is and how passionate love is. Such aggregate behaviors that appear unexpectedly are called “emergent behaviors.” Generally, it is these “emergent behaviors” which are aimed to be understood and changed. To achieve such a goal, the system’s components and how they interact to create these collective behaviors must be understood (Turner and Baker, 2019; Ladyman and Wiesner, 2020).

### 2.3. Non-linearity

Non-linearity is characteristic of systems in which a change in the size of the input will not make a proportionate change in the output (Ladyman et al., 2013).

### 2.4. Edge of chaos

The edge of chaos is a transition space between order and disorder. Think about a pile of sand. If one grain of sand is dropped on top of this pile every second, the pile gradually grows into the shape of a cone. When the pile is at the point that adding even one more grain of sand may dislocate some groups of sand grains, an avalanche is caused. If the motion of the dislodged group is sufficient, a cascade failure in some neighboring groups (subsystems) is generated. CSs naturally evolve toward this state and show a fine balance of sensitivity to perturbations and robust interactions (“self-organized criticality”). Although CS has stable states (attractors) at which it can maintain its stability even if perturbed, it may have unstable states at which a small perturbation can disrupt the system due to subtle interdependencies among its elements. CSs can be sensitive to the slightest disturbance showing the “butterfly effect,” which means that small alternations in the initial conditions may lead to very different results. Sometimes, minimal changes in the environment may entirely change the behavior of the system, known as tipping points, phase transitions, or bifurcations (Lewin, 1999).

### 2.5. Self-organization

There are no external or central organizers in CS. Instead, the “control” of the system is distributed among its agents and integrated *via* their interactions. In ant colonies, the queen does not order ants what to do. Instead, depending only on its local environment and the genetically encoded rules for its responsibility, each ant reacts (Heylighen, 2001).

### 2.6. Hierarchical organization

Complex systems are composed of subsystems (modules) within subsystems within subsystems, which facilitate high levels of functional specificity and the capacity of the system to persist under increasingly difficult conditions. Micro, meso, and macroeconomics in a country are examples of hierarchical organization (Ladyman et al., 2013).

### 2.7. Circular causality

Circular causality includes a continuous and concurrent bottom-up and top-down realization of emergence through self-organization. In this process, macro-level patterns are formed through the micro-level interactions (local-to-global determination), and macro-level patterns set boundaries for micro-level interactions (global-to-local determination) (Witherington, 2011).

### 2.8. Feedback

There is always negative (damping) and positive (amplifying) feedback in CS. In addition to function, feedback can change the structure of the CS (Ladyman et al., 2013; Turner and Baker, 2019).

### 2.9. Robustness

Complex systems are in a state between order and disorder and between segregation and integration. The CSs are stable under perturbations because their organization is distributed and not centrally produced, and their modules are independent while being integrated. For example, eliminating some of the members of a flock of birds does not destroy it (Ladyman et al., 2013).

### 2.10. Hysteresis

In CSs and many natural phenomena, the forward and reverse phase transition pathways are different. For instance, there are distinct temperatures for freezing water and melting ice. In other words, CS may have a memory, and the system is affected by it. Recovery after a critical transition can be more difficult than a simple return to the conditions of pre-phase transition. The history of a CS may thus be important (Fisher and Pruitt, 2019; Ladyman and Wiesner, 2020).

### 2.11. Adaptation

Complex system may adapt and evolve (CAS). These systems can often recover and adapt their prior functionality when their components are damaged or removed, and sometimes they can even improve through loss. This may be achieved through: the capacity to endure across disturbances (robustness), recover to the initial state following a considerable disturbance (resilience), or the capability to alter the system to survive and maintain its function (adaptation). One of the mechanisms that simultaneously leads to robustness, resilience, and adaptation is modularity because each module can both function and change its function without harmfully disturbing the rest of the system (Holland, 2006).

### 2.12. Co-evolution

As the environment that embeds the system changes, systems change to make sure they fit in, and again, the environment changes due to system alterations. CAS are dynamic systems that can learn and evolve from those changes, help affect their environment, predict likely changes, and prepare appropriately.

The brain is one of the most common examples of CAS in the complexity science literature that seems to have all of the CAS features. Accordingly, this article intends to use the science of CSs, neuroscience, and the science of marital relations to start a movement toward understanding the functions of the brain’s CAS in directing marital behaviors (Turner and Baker, 2019).

## 3. Brain’s CAS and marital behaviors

**FIGURE 2:**
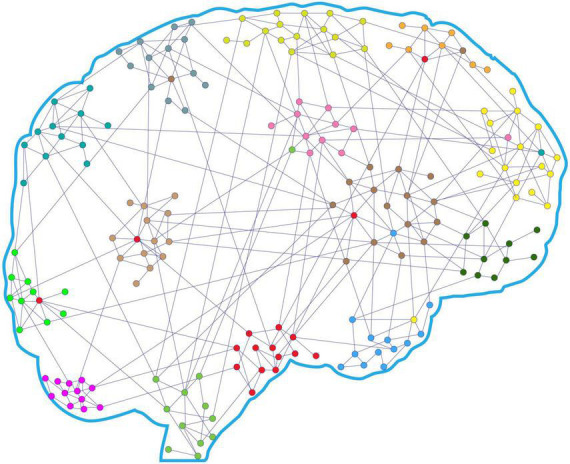
Schematic representation of the brain’s CAS networks. Brain’s CAS is a complex system consisting of a set of brain areas (nodes; colored points) and a set of pairwise connections between them (edges, ties, or links; lines). A specific set of highly correlated nodes and their ties are called networks, modules, or subsystems. The functions of the brain’s CAS involved in marital behaviors are the result of the local integration of brain areas into segregated modules for specific marital constructs (e.g., positive illusion) and the global integration of modules for directing ultimate marital behavior.

A CS consists of items (nodes) and pairwise relations (edges, ties, or links) among the items. The collections of these nodes with their ties are called graphs, networks, or modules (see Figure 2). Decades of functional magnetic resonance imaging (fMRI) and positron emission tomography (PET) experiments have identified functional modules as subsystems in the human brain (including different areas of the brain as nodes (that coactivate during certain types of tasks. Brain functions are the result of the operation of locally segregated modules with specialized functions that integrate as a whole to create perceptions, cognitions, and actions.

As an initial attempt, the fundamental question the researcher is looking to answer is how the brain’s CS is related to marital behaviors. If it can be acknowledged that the brain influences behavior through its mental states and constructs, the source of marital behaviors will be the constructs such as love, empathy, and commitment. Numerous pieces of evidence in the marital relationship literature confirm that these constructs are causally related to positive marital interactions (e.g., Gottman, 2014). On the other hand, studies in social cognitive neuroscience recently show that the psychological constructs such as empathy are the product of emergence that results from the interaction of specific social brain subsystems (Driver et al., 2012).

Therefore, considering that marital relationship makes demands and goals that are so unique and vital, it seems logical for us to have probably evolved specialized neural networks/subsystems (neurologic level), in which mental constructs (psychological level) such as love, positive illusion, and commitment are the product of emergences that arise from the interaction of these subsystems, and ultimately lead to behaviors such as warmth, finding excuses for partner’s transgressions, or reconciliation (behavioral level). To investigate such a possibility, first, essential psychological constructs that can be considered as the reason for positive marital behaviors must be extracted. Citing the literature on marital relationship studies gives us a long list of such constructs. How can a comprehensive and concise list of these constructs and psychological states be obtained?

**FIGURE 3:**
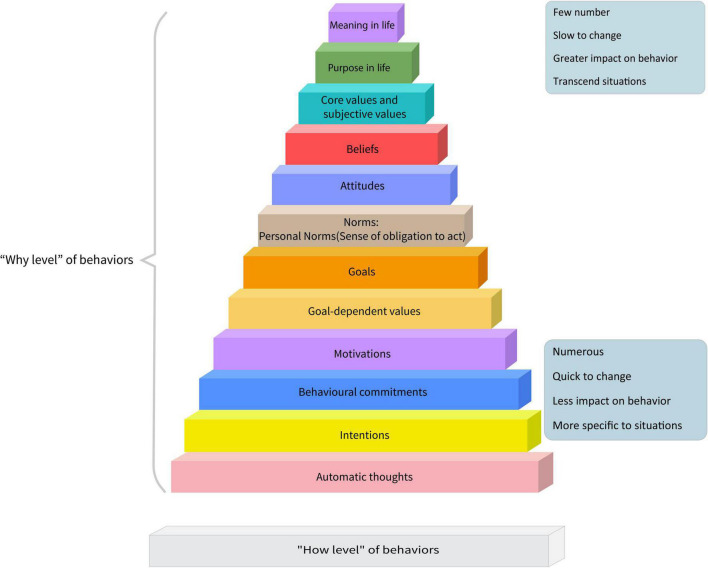
“Why level” cognitive hierarchy. Summarizing previous studies on the causal hierarchy of cognitive constructs that leads to human behaviors, the above model is proposed. Psychological constructs that lead to one’s goal-directed behaviors (including marital behaviors) are organized within a hierarchy of abstractions that are causally related, wherein moving to higher levels is accompanied by a decrease in the number of constructs and an increase in their abstraction and impact on behaviors. Based on different theoretical approaches, some minor displacements in this hierarchy is not unexpected.

According to action identification theory (Vallacher and Wegner, 1987), the theory of planned behavior (Ajzen, 1991), various hierarchical cognitive models (e.g., see Homer and Kahle, 1988; Dietz et al., 2005), and neurological evidence (e.g., see McCall et al., 2012; Spielberg et al., 2013), the representation of all of the people’s goal-directed actions (including marital behaviors) are organized in hierarchically in terms of abstraction that is causally related, wherein going to higher levels of the neuro-behavioral system is accompanied by reducing in the number of constructs and increasing in their abstraction and influence on behaviors. Figure 3 shows a summary of research in this area.

On the basis of action identification theory moving to a higher level of this neuro-behavioral system is equivalent to the “why” of human actions (motivational or “hot” domain: e.g., meaning, attitudes, motivations, and goals) and moving to lower levels is accompanied by the “how” of their actions (cold domain). At the “why level,” the critical determinant is how the individual views the goal object, while at lower levels, strategies and tactics to implement the higher-level demands are selected and implemented. In this system, lower levels are subservient to higher levels. Therefore, “forgiving your spouse for a small mistake” may be represented at higher levels of the system as “I want to protect my children’s mental health (purpose in life), or because I know that their mistake was not intentional (positive illusion)” and at the lowest level as “making a repair attempt by an apology or stop the negative cycle of interactions.”

In order to achieve a comprehensive and concise list of “why level” factors in marital behaviors, a field study has been conducted that had a mixed-method design – part of that was exploratory factor analysis – on 1,670 married individuals (see Supplementary material for details). This research was conducted as part of a series of our 12-year studies and research with the aim of extracting cognitive structures that cause positive marital behaviors from the highest level of the cognitive hierarchy.

According to the majority of approaches in marital relationship studies, marital satisfaction and stability are the two main elements that determine marital trajectories. According to Lewis and Spanier (1979), marriages are divided into four types: satisfied and stable, satisfied but unstable, dissatisfied but stable, and dissatisfied and unstable. In line with many analyses of marital relationship research by leading scholars, it can be concluded that symptoms of dissatisfied couples include more negative behaviors, fewer positive behaviors, and a negative reciprocity pattern (e.g., Driver et al., 2012).

Accordingly, 1,065 married individuals were interviewed to discover the existing themes in their responses to study questions: “(a) what is the source of your positive behaviors during your interactions, conflicts, and difficult moments of the marital relationship? Why do you continue constructive behaviors toward your spouse in such situations? (e.g., humor, empathy, responsiveness, forgiveness, and warmth), (b) When your spouse behaves negatively and destructively, what makes you not reciprocate negatively? (Two questions related to ‘why level’ factors of relationship quality) (c) Despite the conflicts and critical moments in marital life, what factors prevent you from moving toward separation and divorce? (A question related to ‘why level’ factors of relationship stability).” Thematic analysis (Braun and Clarke, 2006) was used to analyze the data obtained from written interviews, and main themes were identified from interviews. Based on the extracted themes, this section, by random selection of 605 married individuals, develops a valid (including Exploratory Factor Analysis) and reliable instrument to provide the possibility of measuring “why level” factors in similar settings (testability) (see Supplementary material for details).

The factors extracted from this study are listed below in Table 1. The findings of this study have strong support in the background of marriage studies (e.g., see Bachand and Caron, 2001), and numerous previous studies show that constructs such as love (Berscheid, 2010), marital satisfaction (Lavner et al., 2016), commitment (Menzies-Toman and Lydon, 2005), positive illusion (Murray et al., 1996), sexual satisfaction (Yeh et al., 2006), religion and spirituality (Day and Acock, 2013), forgiveness (Braithwaite et al., 2011), and other factors extracted from this study directly or indirectly (as motivations, positive attitudes, goals, etc.) play a vital role in the quality and stability of the marital relationship (e.g., see Bachand and Caron, 2001).

**TABLE 1:** The proposed brain’s subsystems/networks involved in marital behaviors.

Subsystems/networks: (inspired by the extracted “why level” factors “a–o”)	The brain regions that make up these subsystems/networks (an instance of references)			Some example of functions related to positive marital behaviors
Love: (a)	Caudate nucleus/putamen, thalamus, VTA, AI, ACC, HPC, occipital cortex, occipito-temporal/fusiform region, angular gyrus/TPJ, dlmFG, STG, and pre-central gyrus (Ortigue et al., 2010)			Anterior cingulate: obsessive thinking about your partner
Empathy, theory of mind, mentalizing, and experience-sharing (mutual understanding): (a)	*Empathy*: dACC-aMCC-SMA, AI, mPFC, dmPFC, vlPFC, VS, vmPFC, mPC, NAcc, VTA, and mOFC			aMCC: cognitive–evaluative form of empathy. Right AI: affective–perceptual form of empathy. Left AI: both forms of empathy mPFC is constantly activated when we think about the internal states of others
	*Mentalizing*: dmPFC, medial frontoparietal network, TPJ, TP, and precuneus			
	*Theory of mind*: dmPFC, pSTS, TP, TPJ			
	*Experience-sharing*: ACC, AI, and PMC (Lieberman, 2007; Fan et al., 2011)			
Marital satisfaction: (b)	VTA, OFC, AI, IFG, BNST, PFC, caudate tail, and decreased activation of SCG (Acevedo et al., 2012b)			OFC: mediates motivation, reward, and action; mediates the effect of cumulative emotional experiences on future behaviors
Positive illusion: (c)	Caudate nucleus, vACC, OFC, vlPFC, dmPFC, reduction of activation of dACC (Song et al., 2018)			Caudate nucleus: gives superiority to the positive attributes of a romantic partner over negative attributes or other social comparisons dACC: suppresses the perception of negative characteristics of a partner
Resilience: (e)	*Emotion regulation and reward systems*: dlPFC, vlPFC, mPFC, HPC, amygdala, insula, ACC, OFC (Carnevali et al., 2018)			PFC: regulates emotional responses by top-down modulating the activation of the amygdala through cognitive/optimistic reappraisal ACC: coping styles and psychophysiological expression of distresses such as heart rate variability OFC: facilitates obtain more resilient behaviors such as flexible adaptation to negative stressors and attention bias toward positive stimuli
Religion and spirituality: (f)	dmPFC, mPC, IFG, rmTG, right precuneus, rsmFG: (theory of mind – ToM-areas), mFG, vlPFC, lmTG, CaG, lFG, left precuneus, left IFG, left STG, STS, FC, amygdala, thalamus (Kapogiannis et al., 2014)			ToM areas: monitor the level of involvement and intent of perceived supernatural agents exemplified by “God.” dmPFC: participates in emotional regulation
Hope: (g)	SMA, PFC, OFC, parts of temporal lobe, parietal lobe, and occipital lobe, cingulum, and HPC (Wang et al., 2020)			SMA: motivates behaviors and producing different ways to solve problems, an optimistic tendency OFC: produces motivation and goal-directed behaviors
Nostalgia: (j)	vmPFC, HPC, SN, VTA, VS, mOFC, X-system specifically the LTC (Oba et al., 2016)			HPC-VS: their co-activation is associated with individual’s nostalgia tendencies’
Sex: (k)	Several nuclei of the hypothalamus and brain stem, basal ganglia, HPC (Coria-Avila et al., 2016)			Hippocampus: After orgasm, the hippocampus facilitate episodic memory consolidation, probably facilitating the crystallization between the experience of orgasm and cues on a partner which lead to pair bonding
Perceived support: (k)	Increased activity in the VMPFC, PCC and precuneus and decreased activity in the dACC, insula, and hypothalamus (Eisenberger, 2013)			These regions trigger down-regulation of physiological response to stress
Commitment: based on studies on neural basis underlying monogamous pair-bonding in mammals and studies on relationship between commitment and executive control: (l)	Medial thalamus, activation of caudate tail, and less activity of medial OFC, NAcc and one part of the SCG (which included BA25), right vlPFC (Xu et al., 2012; Ueda et al., 2018)			rvlPFC: executive control; regulation of people’s interest in engaging in extra-pair relationships, e.g., through “derogation effect” (devaluation of alternative partners and suppression of attention to them) in favor of the long-term goals
Proposed commitment subsystems based on psychological models of commitment (Rusbult et al., 2006)	Models of commitment	Restraining forces	Possible systems/networks	
	*Cohesiveness model – Levinger*	*Attractions*	Marital satisfaction, love, sex, positive illusion, and value based decision-making networks	
		*Barriers*	Religion, reputation, moral, value based, and risky decision-making networks	
	*Investment model – Rusbult*	*Interdependence:* relational identity or “we-ness,” satisfaction and investment size (e.g., presence of children) is high. In contrast alternatives are poor.	Marital satisfaction, love, nostalgia, goal-directed behaviour, and decision-making networks	
	*Tripartite model – Johnson*	*Personal commitment:* interest in partner and wanting to remain in a relationship	Love, marital satisfaction, positive illusion, and value based decision-making networks	
		*Structural commitment:* experiencing constraints that prevent easy dissolution, e.g., social pressure to remain involved in the relationship	Religion, reputation, moral, value based, and risky decision-making networks	
		*Moral commitment:* feeling morally obligated to remain in a relationship	Moral decision-making network	
	*Dialectical model – Brickman*	*Dynamic compatibility with challenges and stresses*	Resilience network	
Forgiveness: (l)	dlPFC, vlPFC, dACC, TPJ/pSTS, mPFC, precuneus and PCC, vmPFC, OFC, IPL, smaller gray matter volume in the right insular cortex and IFG, smaller white matter volume in the left IFG (Ricciardi et al., 2013; Fourie et al., 2020)			dlPFC, precuneus, IPL: respectively, cognitive reappraisal and emotional regulation, perspective taking, empathetic emotional concern for the transgressor. Variations in the activity of these three regions are associated with individual differences in the tendency to forgive.
Goal directed behavior: (e.g., a, h, n, o)	PFC, dlPFC, vlPFC, neocortex, hypothalamus, amygdala, HPC, basal ganglia, OFC (Verschure et al., 2014)			dlPFC: self-regulation in order to achieve goals PFC: plans how to achieve goals
Decision-making: (e.g., d, h, n, o)	OFC, ACC, dlPFC, limbic system, basal ganglia, thalamus, cerebellum, and pons, caudate nucleus, septo-hypothalamic regions, NAcc, vmPFC, IPS, PCC, PPC (Basten et al., 2010; Rosenbloom et al., 2012)			Cost and benefit signals from amygdala and NAcc, respectively, are compared in vmPFC
Stay/leave decision-making: (e.g., d, h, i, l, n, o)	vmPFC, caudate nucleus, and septo-hypothalamic regions, striatum (Heijne et al., 2018)			vmPFC: represents the expected value of the partner
Moral decision making: (e.g., f, l)	vmPFC, OFC, amygdala, TPJ, ACC, AI, PCC, dlPFC, HPC, basal ganglia (Yoder and Decety, 2018)			TPJ: impacts moral judgment by mentalizing and integrating information about the harmfulness of behavior outcomes with information concerning actors’ belief states
Value based decision making: (e.g., f, i, l, n)	vmPFC, dS, dlPFC, PCC, OFC, ACC, VS, PPC (Brosch and Sander, 2013)			vmPFC: plays a key role in representing the personal or subjective value of decision alternatives vmPFC, dlPFC, and OFC: are vital for shaping value judgments in complex situations
Risky decision making: (e.g., d, i, k, n, o)	vmPFC, amygdala and insula, VS, ACC, PCC (Levin et al., 2012)			ACC: continuous evaluation of events and prediction of future events considering desirable or undesirable outcomes
Reputation-based decision-making: (e.g., o)	Striatum, mPFC, vmPFC, OFC, TPJ, amygdala, temporal visual cortex, dlPFC (Izuma, 2012)			mPFC: meta-representation and manipulation of how other individuals think of us Striatum, vmPFC, or OFC: compare the expected reward value (or utility) of one’s reputation with other rewards by using a common currency dlPFC: self-control
Motivational system: (e.g., a, b, f, h, j, k, l, m, n, o)	vlPFC, vmPFC, dlPFC, slPFC, OFC, ACC, VS, PCC, amygdala, insula, hypothalamus, PAG, periaqueductal gray (Pezzulo et al., 2018)			PFC: orchestrates thought and action according to inner goals OFC: plays a role in estimating the value of the stimulus in any state, whether it is a current situation or an estimated future situation mPFC and PCC: representation of goals *via* anticipatory imagery of the future favorable state

FC, frontal cortex; PFC, prefrontal cortex; mPFC, medial prefrontal cortex; vmPFC, ventromedial prefrontal cortex; dmPFC, dorsomedial prefrontal cortex; lPFC, lateral prefrontal cortex; dlPFC, dorsolateral prefrontal cortex; vlPFC, ventrolateral prefrontal cortex; slPFC, superior lateral prefrontal cortex; AI, anterior insula; PI, posterior insula; LTC, lateral temporal cortex; STS, superior-temporal sulcus; pSTS, posterior superior temporal sulcus; mPC, medial parietal cortex; PPC, posterior parietal cortex; ACC, anterior cingulate cortex; dACC, dorsal anterior cingulate cortex; vACC, ventral anterior cingulate cortex; PCC, posterior cingulate cortex; aMCC, anterior midcingulate cortex; VTA, ventral tegmental area; SMA, supplementary motor area; PMC, premotor cortex; TPJ, temporoparietal junction; TP, temporal pole; VS, ventral striatum; dS, dorsal striatum; NAcc, nucleus accumbens; OFC, orbitofrontal cortex; mOFC, medial orbitofrontal cortex; IFG, inferior frontal gyrus; SCG, subcallosal cingulate gyrus; dlmFG, dorsolateral middle frontal gyrus; rmTG, right middle temporal gyrus; rsmFG, right superior medial frontal gyrus; mFG, middle frontal gyrus; lmTG, left middle temporal gyrus; CaG and lFG, bilateral calcarine and left fusiform gyri; STG, superior temporal gyrus; BNST, bed nucleus of the stria terminalis; PAG, periaqueductal gray; HPC, hippocampus; SN, substantia nigra; IPS, intraparietal sulcus; IPL, inferior parietal lobule; X-system, automatic social cognition system. The above-mentioned potential subsystems/neural networks are inspired by the following factors extracted from the exploratory factor analysis of our field study (see Supplementary material) and the background of marital studies on abstract psychological constructs guiding marital behaviors. The extracted factors from exploratory factor analysis: (a) love, mutual fondness, and mutual understanding, (b) marital satisfaction, (c) respect and positive perception of partner, (d) low divorce proneness, (e) resilience and hardiness, (f) religion and spirituality, (g) hope, (h) children, (i) negative attitudes toward conflict and divorce and their consequences, (j) relationship history and long-term investments, (k) fulfillment of needs, sexual gratification, and perceived support, (l) virtues: commitment, tendency to forgive, and willingness to sacrifice, (m) friendship, (n) financial dependence, and (o) reputation and stigmatization.

Previous research shows that these relatively numerous factors impact the quality of marital behaviors. Also, previous studies confirm the interaction of these factors (for example, the interaction of marital satisfaction and forgiveness, see Allemand et al., 2007). Hence, it seems that these factors form a CS. Since these psychological constructs are the product of the brain, which itself is a CS, it seemed reasonable to assume that the networks of the brain, as corresponding neural transcripts, underlie these psychological constructs. Therefore, inspired by these factors and a comprehensive review of the neuroscience literature related to these constructs, the possible neural networks associated with each of these factors were listed (see Table 1). The first column on the left side of Table 1 shows that each neural network is inspired by which of the “why level” factors (a–o) of marital behaviors.

For example, inspired by one of these factors, the tendency to forgive, the brain forgiveness network, consisting of several subsystems whose part of their functions is associated with the tendency to forgive, has been introduced (see Li et al., 2017). Overall, the suggested brain networks in Table 1 include neural networks such as love, marital satisfaction, and positive illusion that are clearly related to marital behaviors and have a strong background in the marriage literature. Other suggested networks include networks such as goal-directed behavior networks and decision-making networks that were paid less attention to in the marriage studies, but some evidence suggests that they are related to marital behaviors (e.g., Ortigue et al., 2010; Heijne et al., 2018), and the results of the field section of the current study highlighted the need of paying attention to these networks. For example, making the proper staying/leaving decisions in a specific marital relationship requires the utilizing of different brain decision-making systems (Heijne et al., 2018) to consider different aspects of this critical decision (e.g., economic and moral aspects).

Regarding commitment, the researcher has tried to present the proposed networks in two ways: first, the neuroscience findings on the neural basis underlying monogamous pair bonding and studies on the relationship between commitment and executive control. Secondly, given that constructs such as commitment are very complex and multidimensional, and there are a variety of theories about them, researcher has tried to consider all the existing theories and then suggested possible neural networks based on the types of commitment. Finally, although there is some information about the neural networks related to some psychological constructs such as friendship (Acevedo et al., 2012a) and psychological needs satisfaction (Reeve and Lee, 2019), it seems that suggestions on the topology of the networks related to these constructs need more studies in the future.

Although most of the neural networks introduced in this article can be considered for relationships other than marital relationships, the current focus is on the marital relationship according to the statistical sample (spouses) and the target research literature.

It is assumed that the proposed networks (subsystems) in Table 1 interact with each other to form a CAS which guides marital behaviors. How this CAS relates to marital behaviors and the quality and stability of the marital relationship will be discussed in the next section of the article under the headings of the assumptions and propositions of BCM.

## 4. Theory of brain complexity and marital behaviors

This theory is a preliminary attempt to understand the CS of brain networks associated with marital behaviors. BCM is based on four underlying assumptions and five propositions derived from complexity theories, neuroscience, and the science of marital relationships. The following sections present and discuss these assumptions and propositions.

### 4.1. Assumptions

#### 4.1.1. Like other human behaviors, the main origin of marital behaviors is the brain

Although contextual factors play an important role in marital behaviors as triggers or catalysts – and their role is considered in Proposition 5 – it is the brain that determines the final and adaptive behavior considering these contextual factors and depending on the resources it has (its unique structure and function in each individual), as two different people in the same context can behave differently.

#### 4.1.2. The human brain influences marital behaviors and, ultimately, the quality and stability of a relationship through its psychological constructs such as love, marital satisfaction, commitment, empathy, etc.

In addition to the findings of current field research on “why level” factors of marital behaviors, the history of marital relationship science are full of evidence that shows that these abstract constructs may be considered the source of positive marital behaviors and the quality and stability of the marital relationship. For example, previous studies suggest that cognitions and behaviors that prevent the decline of relationship quality can possibly be motivated by fundamental constructs such as commitment (Menzies-Toman and Lydon, 2005) and marital satisfaction (Lavner et al., 2016). Similarly, marital therapists and ex-spouses often remark on the “death of love” as the most important reason for divorces or separations (Gigy and Kelly, 1993; Whisman et al., 1997).

#### 4.1.3. The mentioned psychological constructs are created as a result of brain functions and their input (e.g., interactions with the spouse and learning)

All disciplines related to neuroscience and psychology concur that the mental states that lead to one’s behaviors are created by the specific functions of the brain. In addition, all of these disciplines acknowledge that the function of the brain is influenced by external inputs in order to provide an adaptive response to the environment.

#### 4.1.4. The brain follows the rules of complex adaptive systems and biological rules of the brain

As mentioned earlier, the brain is perhaps the best example of a CAS in the literature on complexity science (e.g., see Ladyman et al., 2013). The second part of this assumption can be taken for granted.

Before introducing BCM’s propositions, it is important to get acquainted with its main concepts.

### 4.2. Concepts

#### 4.2.1. Brain’s CAS involved in marital behaviors

The set of all subsystems of the brain that, as a CAS, directs marital behaviors.

#### 4.2.2. Subsystem

The network that is composed of different areas of the brain and forms a functional module to participate in the creation of one of the marital constructs (e.g., the positive illusion subsystem; see Table 1).

#### 4.2.3. Marital constructs

Abstract psychological constructs such as love, marital satisfaction, commitment, and positive illusion that can trigger and affect marital behaviors.

#### 4.2.4. Marital behaviors

Behaviors that play a role in the quality and stability of the marital relationship include positive marital behaviors, such as empathetic behaviors, and negative marital behaviors, such as negative reciprocation.

#### 4.2.5. The quality of the marital relationship

The quality of the marital relationship means the level of marital satisfaction of the couple or the happiness of the marriage.

#### 4.2.6. The stability of the marital relationship

The stability of the marital relationship is equivalent to the degree of stableness and durability of marriage.

#### 4.2.7. Quality of information and inputs

The quality of interactions with the spouse, learning, new skills, and information about the context of the marital relationship (e.g., socio-economic conditions, stressors, the existence of alternatives, and barriers) that enters the brain.

#### 4.2.8. Structural architecture

Structural architecture refers to the quality of the structure of the brain’s networks involved in marital behaviors and their unique composition in one person.

#### 4.2.9. Functional architecture

Functional architecture is the quality of functional interaction of the brain’s networks involved in marital behaviors (e.g., the quality of interaction between marital satisfaction and forgiveness networks) and their unique composition in one person.

#### 4.2.10. Quality of recruitment of networks

The quality of using a brain’s networks efficiently in favor of the marital relationship.

### 4.3. Propositions

#### 4.3.1. The marital constructs such as love, positive illusion, marital satisfaction, etc., are the product of emergences that result from the function of a network of specific areas of the brain (subsystems) and/or their interaction with other brain networks (proposition of modularity/segregation and emergence)

**FIGURE 4:**
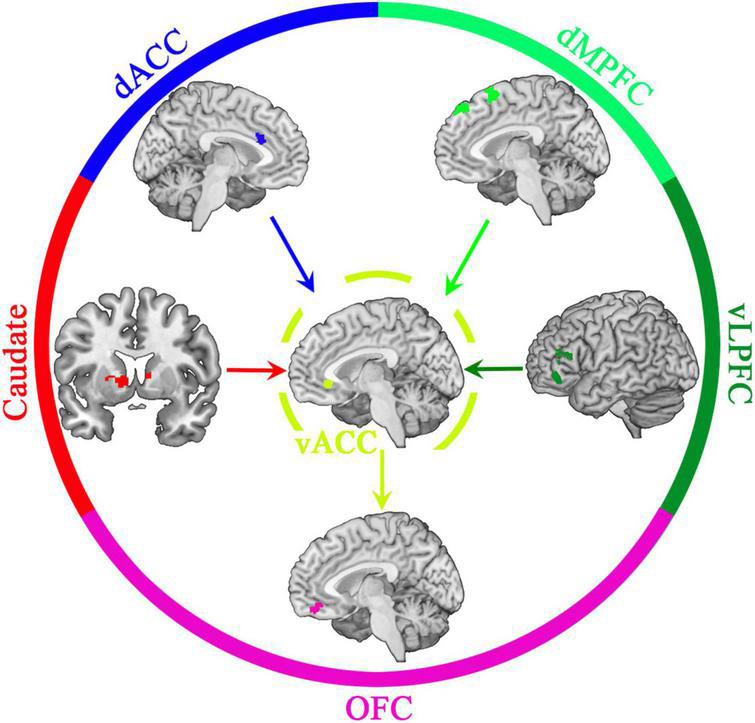
Positive illusion (seeing one’s partner generously) emergence in the brain and its subsystem. The caudate nucleus highlights the positive features of a romantic partner compared to negative features or other social comparisons. Decreased dACC activation suppresses the perception of negative characteristics of the partner. In the meantime, the dMPFC and vLPFC lessen the prominence of tempting rivals. Then the vACC intensifies the distinction between the favorite social features of intimate people and other people. Finally, when information is transmitted to the OFC, the weight of a partner’s positive and negative characteristics is re-assessed, reinforcing biased, subjective values (e.g., positive illusion) about the romantic partner—reprinted with permission (Song et al., 2018).

Today, primarily due to the success of cognitive neuroscience models, researchers know that people’s intellectual abilities emerge from the operation of neural systems that are instantiated by specific brain networks (see Driver et al., 2012). In the context of marital behaviors, is there any evidence for the phenomenon of emergence in the brain networks? Numerous studies confirm the role of positive illusion as one of the important constructs in triggering positive marital behaviors (Murray et al., 1996). Does the brain have a specific network or subsystem that emerges positive illusion? Song et al. (2018), reviewing the literature on relevant brain networks and areas involved in positive illusion, demonstrated how positive illusion might emerge from the interaction of certain parts of the brain (Song et al., 2018; see Figure 4).

In this functional network, the caudate nucleus activation gives salience to the positive attributes of a romantic partner over the negative ones or other social comparisons. The dACC (see the footnote of Table 1 for abbreviations) has been linked to conflict monitoring, error detection, and social exclusion. Thus, decreased dACC activation suppresses the perception of a partner’s negative attributes. Increased vACC activation could boost the discrimination of favorable social qualities between beloved and non-intimate individuals. Simultaneously, the dMPFC and vLPFC could lower the superiority of charming rivals. Lastly, when information is transferred to the OFC, the evaluation of a partner’s positive and negative information is redistributed, cementing biased, subjective values (e.g., positive illusion) (see Figure 4). It appears that different areas of the brain that are active in this network have a specific job, just as distinct groups of workers in an ant colony, but their collective end product is completely different from the sum of their individual works. Notably, the above-mentioned regions of the brain do not function independently but interact with each other (synchronicity).

Similar patterns can be found in recent neuroscience findings for some other psychological constructs related to marital behaviors, such as forgiveness (Fourie et al., 2020), empathy (Zaki and Ochsner, 2012; Driver et al., 2012), romantic love, attachment, moral judgment (Driver et al., 2012), resilience (Yao and Hsieh, 2019), motivation (Spielberg et al., 2013), decision making (Basten et al., 2010), and reputation-based decision-making (Izuma, 2012).

#### 4.3.2. The mentioned psychological constructs in circular causality processes are the sources and products of brain downstream processes and inputs/outputs such as marital behaviors

In the history of marital relationship studies, we are left with a seeming inconsistency: love is both blind (top-down causation) and deep-rooted in the real world (bottom-up causation). Previous observation reasonably suggests that in intimate relationships, reality and illusion go together in the creation of a happy and stable relationship. However, the mechanism of this process has remained an unsolved conundrum.

A similar question arises about the origin of empathy in interpersonal relationships. A brief description of the circular process of empathy formation at high levels of brain hierarchy and the subsequent realization of empathy at the behavioral level can be enlightening in this regard. Neurological studies indicate that the activation of an observer’s empathetic response is usually triggered by external cues, yet it has long been argued that contextual assessment (such as the relationship value), cognitive processes, and top-down control are key components of human empathy (empathy bias). This meta-cognitive level is regularly updated by bottom-up information and, conversely, controls the lower levels *via* top-down feedback, which makes the individual less dependent on external clues (Singer and Lamm, 2009).

In such circular causation, lower brain-mind functions are integrated (see emergence processes in proposition 1), considering higher-order mind-brain constraints (e.g., empathy bias, the blindness of love, biased selection, amplification, or inhibition of inputs), which then provide top-down regulatory control over downstream processes in brain and behaviors such as empathetic response (see Panksepp and Panksepp, 2013, for the details of processes for empathy).

In addition to empathy, there is ample neurological and psychological evidence of circular causation for other constructs such as love, marital satisfaction, resilience, positive illusions, and commitment (e.g., see Lavner et al., 2016; Ioannidis et al., 2020).

#### 4.3.3. The brain’s subsystems form a complex adaptive system for guiding marital behaviors (proposition of collaboration or integration)

At the psychological level, there is significant evidence for the existence of a complex relationship between psychological constructs related to marital behaviors and their interaction with each other. For example, Chung (2014) showed that insecure attachments negatively affect marital satisfaction and forgiveness through a lack of empathy. Also, in the field of marital interventions and couple therapy, the failure of marital interventions due to the interference of higher-order cognitive constructs such as marital satisfaction and spouses’ affect has astonished researchers (see Gottman and Tabares (2018).

Signs of the presence of a complex neural system related to marital behaviors and interaction between its subsystems can be seen in experiments conducted to investigate the effect of holding a spouse’s hand compared to a stranger or no hand at all when spouses are threatened with an electric shock. Coan et al. (2006) found that holding the spouse’s hands in a threatening situation results in a pervasive decrease in the activation of threat-related neural networks, while this attenuation is more limited in the case of strangers. More interestingly, with the increase in the quality of the marital relationship, the calming effect of holding the spouse’s hand increased on neural threat responses (superior frontal gyrus, right anterior insula, and hypothalamus). Similarly, Coan et al. (2017) found that under the threat of shock, higher perceived social support is associated with lower neural activity in an extended networks related to regulatory self-control, salience, and vigilance, but only when holding a partner’s hand and not friends. Similar findings have been reported in psychological (Horn et al., 2019; Liu et al., 2021; Jakubiak, 2022) and neuroscience (Younger et al., 2010; Morriss et al., 2019) studies.

As an additional example, it can be useful to consider the connections of marital satisfaction networks with other neural networks and some of the possible effects of these interactions on marital behaviors. In the context of the romantic relationship and at the behavioral level, both trait forgiveness and relationship satisfaction are related to forgiveness behavior. When all three variables are considered at the same time, the interaction of lower levels of marital satisfaction of the hurt partners with their trait forgiveness may reverse the benefits of trait forgiveness; high trait forgiveness in satisfying relationships leads to the possible episodic forgiveness, but in dissatisfying relationships, it results in the least possible episodic forgiveness (Allemand et al., 2007).

To clarify such observations, considering the connections between the marital satisfaction network and the forgiveness network can be informative. Acevedo et al. (2012b), found that marital satisfaction is significantly correlated with activation in several brain areas, including the AI (associated with empathy); the VTA (reflecting reward and motivation); the OFC (related to the evaluation of rewards); the IFG (related to the mirror system), the BNST (related to stress control); and the PFC (related to affective regulation). Among the mentioned areas, PFC and OFC, as the two “connector hubs” (nodes that belong to several intersecting networks and link multiple modules) of the brain’s functional network (van den Heuvel and Sporns, 2013), overlap with the forgiveness network. Several other of these areas overlap with empathy, emotion regulation, and mentalizing networks that indirectly play a vital role in the forgiveness process. This integration of information may lead to an adaptive decision about forgiving the transgressor partner depending on the context and temperature of the relationship. Acevedo et al. (2012b) believe that these findings exemplify how perceived relationship satisfaction “evokes brain systems that influence choices and guide behaviors that may serve to promote relationship well-being and stability (p. 26).”

The marital satisfaction network overlaps with the vast majority of marital networks (see Table 1). Such a broad impact on the functional networks of the brain may explain phenomena such as sentiment override (Weiss, 1980), which means people, interpret their spouses’ behaviors as consistent with their current level of marital satisfaction rather than the partner’s actual behaviors. Similar to what came about marital satisfaction, there is ample evidence that the activity of brain regions supporting empathy is not fixed but may be modulated by contextual appraisal or personal motivations such as liking (see Weisz and Zaki, 2018, for review). Acknowledging the overlap of the empathy network and the other brain networks proposed in Table 1 can explain such findings. In this regard, social neuroscience findings indicate that there is a complex relationship between empathy and morality, and neural networks of empathy and morality meet each other in the vmPFC as a critical hub (Decety and Cowell, 2014).

#### 4.3.4. The quality of marital interactions and the quality and stability of the marital relationship are influenced by the quality of the structural and functional architecture and the recruitment of the brain’s CAS involved in marital behaviors

The field is just at the beginning of the road of the neuroscience of marital behaviors, and part of a few existing findings in this area are usually correlational, and causal inferences need further research. However, there still are valuable findings that show the existence of individual differences in the mentioned domains and their possible impact on behaviors related to the marital relationship. For instance, Ma et al. (2022a) demonstrated that husbands’ differences in large-scale neural networks when they process their wives’ marital interactions may predict their variability in marital relationship quality 13 months later. Xu et al. (2012) found that the quality and stability of marriage until the 40th month of the relationship is predicted by lower activation of medial OFC, right accumbens, and right subcallosal cingulate during the early stages of love (Xu et al., 2012). Ueda et al. (2018) showed that VLPFC activity, which is implicated in executive control, can predict the stability of monogamous relationships in long-term relationships.

Recently Ma et al. (2022b) compared resting-state functional connectivity (FC) of couples with their FC during social information processing (watching relationship-specific and general emotional stimuli) and examined the relationship between their relationship quality and reconfiguration efficiency (these two FC similarities) 13 months later. Results indicated that the more easily wives can shift from a resting state to a relationship-specific information processing state (higher reconfiguration efficiency), the higher relationship quality they experienced 13 months later. The previous research shows that the pattern of brain function of people who have been married for an average of 21 years while viewing the facial images of their spouse is different than when they view the image of a close long-term friend, a highly familiar acquaintance, or a low-familiar person. The former involves recruiting areas of the brain related to reward and attachment. Also, the level of functioning of these parts of the brain was correlated with the love scores, its type and frequency of sexual activity (Acevedo et al., 2012a).

In addition to the neurological differences directly associated with the quality and stability of the marital relationship, neurological differences related to psychological constructs that may be the source of marital behaviors have also been observed. For example, structural imaging studies discovered that affective empathic abilities correlate negatively with gray matter volume (GMV) in the precuneus, inferior frontal gyrus (IFG), and anterior cingulate (Banissy et al., 2012).

Fincham et al. (2007) believe that the differences in the quality and stability of marriages are due to the existence of constructs such as forgiveness, commitment, sacrifice, and sanctification, which trigger transformational and homeostatic processes in the relationships, and therefore reduce negative retaliation responding across repetitive cycles of couples’ interactions (Fincham et al., 2007). It is likely that the individual differences in these internal resources are equivalent to the differences in the quality of structure, function, and recruitment of neural networks associated with these psychological constructs. For example, Li et al. (2017) showed that there are associations between higher tendency to forgive (TTF) scores and larger GMV in the areas of dlPFC and smaller GMV in the areas of the IFG and right insular cortex. In addition, higher TTF scores are associated with the smaller white matter volume in the left IFG.

Theory of brain complexity and marital behaviors proposes that, in addition to structure and function, the quality of recruitment of brain networks also affects the quality of marital interactions and their consequences. For example, neurological studies show that the quality of theory-of-mind (ToM) network recruitment can determine the outcomes of empathy and mentalizing exercises. Partners might consider a wide range of actions representative of ToM (e.g., nodding), while there is neurological evidence that suggests only in certain cases do such behaviors are indicative of actual involvement of brain regions that are related to the ToM (e.g., nodding and considering the point of view of partner, compared with nodding while discretely checking phone notifications). Dodell-Feder et al. (2016) found that only high neural selectivity in the left temporoparietal junction (LTPJ) and precuneus (the neural network supporting ToM) for beliefs versus physical qualities of the partner is significantly related to the wellbeing of the partner, up to two days after a conflict. Remarkably no association was found between perceived understanding and subsequent partner’s wellbeing in the case of low activation of LTPJ and precuneus for belief information.

Correspondingly, an fMRI study on forgiveness showed that costly apologies (as a signal of conciliatory intention) significantly activate the ToM network (i.e., precuneus, bilateral TPJ, and mPFC), unlike non-costly apologies (i.e., only saying “sorry”), and non-apologies. Importantly, Ohtsubo et al. (2018) reported that no significant difference in brain responses to non-apology controls and non-costly apologies was observed. Also, for forgiveness in which people experience emotional concern for their transgressors, activation in the inferior parietal lobule is selective, a process that facilitates empathy with the offender (see Ricciardi et al., 2013, for review).

#### 4.3.5. The quality of information and inputs received and processed by the brain’s CAS involved in marital behaviors affects the quality and stability of the marital relationship

According to BCM, inputs, including the information about the quality of a partner and couple’s interactions (equivalent to what behavioral theories emphasize), the value of a relationship, alternatives, and barriers (social exchange theory), the fulfillment of needs quality (attachment theory) or the stressors (crisis theory and VSA model) is monitored, stored and updated in the brain networks and ultimately affects the spouses’ interactions and the quality and stability of their relationship.

In this regard, neurological studies indicate that the partner and relationship value is upregulated in response to the partner’s pro-relationship behaviors, and the mOFC – which as a hub, contributes to various systems – is involved in this recalibration process (Ohtsubo et al., 2020). Furthermore, the partner’s positive reciprocity robustly engages the ventral striatum and OFC (Phan et al., 2010). This reciprocity signal in these areas emerges only in reaction to partners who have consistently returned the investment and do not exist for partners who do not have a reputation for reciprocity. A previous study has found that having a greater amount of positive experience in a relationship before a trust breach helps with the recovery of trust—even after trust breaches—by activating the automatic social cognition neural system (or X-system: habitualized, more automatic, and less reflective system) (Schilke et al., 2013). Similarly, higher perceived support from the partner activates specific areas of the brain, which simultaneously trigger down-regulation of physiological response to stress (Eisenberger, 2013), and according to the VSA model and crisis theory, any resource that can act as a buffer against stress can improve the quality and stability of a marital relationship (Karney and Bradbury, 1995).

In addition, if concurrent marital satisfaction is considered a significant representative of the quality of marital interactions (Gottman and Krokoff, 1989), different brain systems associated with empathy, emotion regulation, decision-making, reward and motivation, and stress control can be evoked by marital satisfaction (Acevedo et al., 2012b) which ultimately can affect the quality of marital behaviors, conflict management, and relationship wellbeing and stability. This is consistent with the VSA model of marriage, which believes that the quality and stability of a relationship are directly influenced by relationship satisfaction, which interacts with the coping processes or problem-solving skills of the partners (Karney and Bradbury, 1995).

Finally, due to the subjectivity of perceptions and top-down control of the nervous system, it is believed that (in selecting, amplifying, modifying, or inhibiting inputs), the effectiveness of the inputs (including couple interactions) depends on the quality of the architecture of the individual’s neurocognitive organization.

## 5. BCM and existing theories

At the same time that BCM is consistent with existing theories, it has the potential to address part of the complexities of marital behaviors beyond the scope of extant theories. For example, unlike behavioral models, BCM considers the underlying neurocognitive sources of marital interactions (e.g., positive illusion, marital satisfaction, and resilience). BCM can also explain the phenomenon of marital self-repair as one of the CAS’s characteristics (self-organization and adaptation). Fincham et al. (2007) suggest that such self-repair processes can be realized *via* “positive, meaning-related constructs” such as forgiveness, sacrifice, commitment, and sanctification (Fincham et al., 2007). In line with this assumption, BCM can explain this process by introducing a complex neurocognitive system part of its subsystems is forgiveness, commitment, religion, and spirituality. Also, BCM has the potential to explain why some couples with negative interaction patterns continue their relationship despite long-term conflicts. In situations where the subsystems of marital satisfaction and love are inactive because of successive conflicts or the low rate of positive interactions, the presence of other subsystems, such as hope, resilience, religiosity, etc., can stabilize the marriage (robustness, resilience, and adaptation of CAS).

Theory of brain complexity and marital behaviors also has this strength of behavioral theory that describes marriage as a dynamic phenomenon and believes that marital quality and stability are justified by each satisfying interaction, making further satisfying interactions more likely. As mentioned earlier, parts of the brain networks involved in marital behaviors regularly update and recalibrate the value of the partner and relationship based on information received every day from the partner’s behaviors. In this respect, BCM and behavioral theory provide explanations for the dynamic change in the marriage that are not considered in the perspective of social exchange theory. From the perspective of social exchange theory, factors such as religion, commitment, and children are considered barriers to marital dissolution just at the moment of deciding to maintain or dissolve the marital relationship. Whereas in the BCM, these factors, in addition to stability, can be sources of satisfaction and quality of the relationship and motivating factors to create positive behaviors in every moment of the marital relationship and therefore have a dynamic role.

Furthermore, BCM, in contrast with social exchange theory, has the potential to explain how different people may behave differently in low reward conditions as a result of psychological characteristics and constructs such as resilience. Lawler and Thye (1999) believe that “the actors of exchange theories are normally viewed as individualistic, instrumental, and unemotional, whereas those of emotion theories are socially oriented, expressive, and emotionally deep and complex. The former is driven by reason, the latter by passion (Lawler and Thye, 1999, p. 33).” In contrast, BCM considers the human as a multidimensional being who simultaneously has a passion (e.g., love) and reason (e.g., economic decision-making), and the integrated information from the interaction of all the neural networks forms the final emerged marital behaviors and decisions. In this regard, fMRI studies performed to examine activated brain regions when deciding to maintain or dissolve a relationship indicate that the remaining decision goes above and beyond the objective and economic value of staying (Heijne et al., 2018).

Theory of brain complexity and marital behaviors also seems to have the capacity to address the gap in crisis theory and the VSA model regarding the details of adaptation resources, adaptive processes, and personal characteristics, which act as buffers against stress. It seems that the quality of structure, function, and recruitment of neural networks introduced in the BCM, the psychological construct emerging from these networks, and their potential cognitive and behavioral implications are theoretically related to adaptive processes. In addition, according to Hobfoll (1989), everything that people value can be a resource for adjusting to stressful life events and increasing compatibility with critical situations. Therefore according to the previous studies (e.g., see Eisenberger, 2013; Basińska and Sołtys, 2020), resilience, hope, perceived support, religiosity, and spirituality directly, or anything that can activate the motivational system, goal-directed behavior system, and/or decision-making systems (such as having children), directly or indirectly, have the potential to play a significant role in coping with stress. Also, BCM, by including love, sex, perceived support, and marital satisfaction subsystems, addresses the concerns of attachment theories, which emphasize the satisfaction of needs and sexual gratification as determinants of stability and quality of the relationship.

Finally, psychological capitals such as hope have no decisive role in existing theories of marital relationship, whereas hope for future satisfaction is a more important factor in determining the quality and stability of marriage than current relationship conditions (Baker et al., 2017). By emphasizing important constructs and resources such as hope, forgiveness, religion, spirituality, and resilience, BCM brings them from the margin to the center.

## 6. Testability of the theory: Replicability and falsifiability

Replicability and falsifiability are two of the main criteria of a scientific theory (e.g., see Earp and Trafimow, 2015). A study is replicable when a replication study of it (utilizing sufficiently similar methods under sufficiently similar circumstances) can be carried out (Peels, 2019). A scientific theory must be falsifiable in the sense that it can be logically contradicted through an empirical test utilizing existing technologies (see Earp and Trafimow, 2015, for review).

It seems that the replicability of BCM can be discussed on three levels. Level (a): the field part of the present study, which led to the extraction of the list of higher-order cognitive factors (e.g., love and commitment) effective on marital behaviors. Level (b): the attribution of these factors to the corresponding neural networks. And level (c): all of the neurological–psychological studies mentioned by other researchers that potentially support this theory and its propositions. Details of the methodology for replication at the (a) level are available in Supplementary material of the article. In addition, the inductive approach in the field part of the study was completed simultaneously by the deductive methods (see Supplementary material for details) to align with the repeated findings in the literature of marital relationship studies. Therefore findings in the field section of the study are not surprising or new. Different types of studies conducted in the history of marriage studies in a relatively similar way to the field part of this research (e.g., see Bachand and Caron (2001); and Asoodeh et al. (2010), as review studies (Billingsley et al., 2005; Karimi et al., 2019), or research which have studied each factor independently (Murray et al., 1996; Menzies-Toman and Lydon, 2005; Yeh et al., 2006; Berscheid, 2010; Braithwaite et al., 2011; Day and Acock, 2013; Lavner et al., 2016), have obtained similar and consistent findings to field section of present study indicating the validity and replicability of the results.

Regarding the replicability at level 2 (attribution of psychological constructs to neural networks), it seems that other researchers also act similarly regarding the neural networks introduced in the case of constructs such as love, marital satisfaction, and positive illusion. However, the replicability of how neural networks are introduced in the case of constructs such as commitment is debatable. In level 3, the available neuroscience evidence about some neural networks (for example, empathy and love) has reached a significant degree of consistency, but about others, it raises the possibility that future research will cause changes in the topology of the networks presented in this article.

In order to test and falsify BCM theory and its propositions, it is feasible to utilize MRI studies, fMRI studies, brain lesions studies, repetitive transcranial magnetic stimulation (rTMS) of a specific area of the brain which interferes with its activity and provides a causal inference (virtual lesion), or other neurological instruments and assessments and analyzing and relating the results obtained from these methods with behavioral observations of couples or psychological assessments (such as measuring their love, empathy, tendency to forgive, marital satisfaction, etc.) using valid and reliable questionnaires. In order to falsify BCM theory, it should be shown that the mentioned neural networks (e.g., empathy network) are unrelated to marital behaviors (e.g., empathy with a spouse). It should also be shown that there is no interaction between the neural networks mentioned in BCM theory (for example, between the empathy network and the moral decision-making network – falsifiability of the theory and proposition 3). It seems that the evidence available in the field of marital relationships or similar settings not only indicates the testability of this theory but also confirms it. For example, refer to the studies mentioned in Table 1 for the possibility of investigation of the relationship between the mentioned neural networks and behavior. Also, the studies conducted to investigate the interaction between the mentioned neural networks and their effect on behavior are available in similar settings (e.g., Decety and Cowell (2014).

To falsify proposition 1, it should be shown that the collective behavior of the agents of a specific neural network (for example, in the love or moral decision-making system) is unrelated to their love, quality of marital interactions or spouses’ decisions, or that the function of individual elements of these networks is not different from the collective behavior of networks as a whole (definition of emergence; Holland, 2002). For example, it should be indicated that real or virtual lesions (by rTMS) in one or a number of nodes of these networks do not affect the networks’ collective behavior. Such testability is feasible and has been experienced in similar or related contexts, and the findings are in line with confirming this proposition. For instance, Knoch et al. (2006) showed that virtual lesions (by rTMS) in dlPFC influenced the participants’ decisions to accept their partner’s unfair offers. Damasio et al. (1990) have documented the case of a patient (E.V.R.) who had a happy and stable marriage and a successful professional life prior to his orbitofrontal lesion because of meningioma and its inevitable surgery. After the surgery, his social behavior changed profoundly; he divorced twice and went bankrupt in his professional life (note Table 1 and the extensive roles of the OFC as a hub). Also, the study of patients with real brain damage in different areas of the brain related to empathy shows that changes in empathy as an integrated construct differ depending on the location of the brain lesions (Shamay-Tsoory et al., 2009). It means that empathy, as a whole construct, is the product of the integrated function of all elements of the empathy network and is different from the function of individual elements or a limited number of them.

To falsify other propositions, it should be indicated that effective environmental inputs such as positive marital behaviors or psychological interventions have no effect on this CAS (falsifiability of propositions 2 and 5) and this possible obtained effect at the neurological level does not extend to marital behaviors at the same time (falsifiability of proposition 2). It seems that such experiments have been done, and it shows that these propositions can be testable/falsifiable, and the results obtained so far confirm these propositions. For instance, one of these studies shows that satisfying sexual activity and its frequency in marital relationships is associated with the activation of cortical areas that mediate empathy, self−other processes and complex thinking, as well as subcortical areas which support basic physiological and motivational processes (Acevedo et al., 2019). Similar findings regarding the significant and positive effect of some simple and short-term learning experiences on the empathy and function of parts of the empathy neural network (anterior insular cortex) (Hein et al., 2016) are available.

It should also be shown that the quality and stability of the marital relationship are unrelated to the structure, function and type of recruitment of this CAS and its neural networks (falsifiability of proposition 4). Recent studies suggest the possibility of such assessments. In this regard, some studies show that differences in the structure, function and type of recruitment of brain networks are associated with different outcomes in the quality and stability of marital relationships (Xu et al., 2012; Dodell-Feder et al., 2016; Ueda et al., 2018; Ma et al., 2022a,b).

## 7. Practical implications

Theory of brain complexity and marital behaviors can have therapeutic, educational, and research implications in the field. In other words, the utilization of the brain’s and CSs language in the design of interventions and therapies can improve achievements in this area. For instance, discovering the details of the structure and function of the subsystems of the brain’s CAS involved in marital behaviors (e.g., positive illusion: see Figure 4 and its caption) can lead to the design of relevant interventions and therapies. In addition, this approach can also trigger research on developing researchers’ knowledge about the structure of the brain’s CAS involved in marital behaviors. Also, discovering the connections and interactions between the brain’s subsystems [e.g., between marital satisfaction and forgiveness (Allemand et al., 2007) or between moral decision-making and empathy (Decety and Cowell, 2014)] can lead to considering these interactions in designing interventions and therapies. Heino et al. (2021) believe that behavior change interventions without considering the rules governing CSs “is akin to attempts to work against gravity, which pulls a ball to the bottom of a valley (p. 4).”

Furthermore, today the vast majority of the interventions and therapies in the field have mainly focused on conflict resolution and communication skills. However, longitudinal studies indicate that this approach has not led to promising results (Karney and Bradbury, 2020). Whereas, based on BCM, it seems that paying attention to constructs such as forgiveness, hope, resilience, spirituality, religiosity, and morality, is necessary while considering communication and conflict management skills to improve marital relations. In this regard, future interventions and therapies may have the potential to utilize neurobehavioral networks that already exist in people (e.g., people’s spirituality) and have not yet been tapped to benefit the relationship, or improve the structure and function of the brain’s CAS in favor of the relationship, relying on the plasticity of the social brain (Valk et al., 2017). BCM also raises the potential value of research on the practicability of using non-invasive neurological interventions such as transcranial alternating current stimulation (tACS) to improve marital relations, which its practicability has recently been discussed at a theoretical level (Liu et al., 2019).

## 8. Conclusion

This article, relying upon the science of complexity, neuroscience, a vast body of evidence in the history of marital relationship studies, and significant qualitative and quantitative findings from the study of 1,670 married individuals, presented the BCM. In the initial stages of the research, the author sought to find higher-order cognitive factors as “why level” factors of marital behaviors. The findings of the field section of the study resulted in a set of well-known cognitive constructs in the research literature on marital relationships, such as love, commitment, marital satisfaction, and other motivational factors. Analyzing the complex relationships between these factors, their complex relationships with marital behaviors, the existence of hierarchy in this cognitive-behavioral organization, and the interaction between these factors suggested the possibility of the existence of a CS consisting of these factors. Since these factors and their related processes are the product of the brain and the brain is a CS, the researcher investigated the possibility that this complex cognitive-behavioral system is an abstract version of a CS consisting of specific neural networks. Therefore the study of the neuroscience literature on the neural networks attributable to these extracted psychological factors led to the presentation of the BCM theory.

Although there is a wide background of knowledge on the relationship between the brain and behavior in psychology and neuroscience literature, BCM, for the first time, explained the relationship between the brain and a complex behavior such as marital behavior from the perspective of complexity science. Being on the rails of CSs will cause fundamental changes in the science of marital relationships because the laws governing CSs fundamentally differ from the laws of simple or even complicated systems and reductionistic perspectives.

It seems that integrating the science of complexity, neuroscience, and the science of marital relationships can lead to the emergence of novel capabilities that facilitate the evolution of marital relations science. Future findings in neuroscience and the science of marital relationships can lead to the completion and adjustment of some of the networks presented in this article. Future research will be utilizing BCM in the fields of education, prevention, and therapy. This article may inspire interdisciplinary studies of marital relationships, CSs, and neuroscience.

## Data availability statement

The raw data supporting the conclusions of this article will be made available by the authors, without undue reservation.

## Author contributions

The author confirms being the sole contributor of this work and has approved it for publication.
