# Supplementary material for: Theory of brain complexity and marital behaviors: The application of complexity science and neuroscience to explain the complexities of marital behaviors
Source: Front Hum Neurosci. 2023 Mar 7;17:1050164. doi: 10.3389/fnhum.2023.1050164 (PMC10027753; doi:10.3389/fnhum.2023.1050164)

Supplementary file: The field study

The Extraction and Measurement of "Why Level" Factors of Marital Behaviors

1. Introduction

This section is a brief report of an attempt toward the extraction and quantification of "why level" factors of positive marital behaviors. According to the majority of approaches in marital relationship studies, marital satisfaction, and stability are two main elements that determine marital trajectories. According to Lewis and Spanier (1979), marriages are divided into four types: satisfied and stable, satisfied but unstable, dissatisfied but stable, and dissatisfied and unstable (Lewis & Spanier, 1979). On the basis of many analyses of marital relationship research by leading scholars, it can be concluded that symptoms of dissatisfied couples include more negative behaviors, fewer positive behaviors, and a negative reciprocity pattern (Driver, Tabares, Shapiro & Gottman, 2012).

Hence, the primary aim of this section was to the extraction of the fundamental factors ("why level" factors) that spontaneously lead to fewer conflicts and negative reciprocity, more positive marital behaviors, stabilize the relationship, and ultimately create a happy and stable marriage. Furthermore, this section develops a valid and reliable instrument as a secondary aim to provide the possibility of measuring "why level" factors in similar settings. Meanwhile, measurement of the "why level" factors may be an important data source for assessments, designing interventions, and treatment planning.

2. Methods

2.1. Study setting and design

Figure 1 shows the study flow diagram. A mixed-method design combining qualitative and quantitative procedures was utilized. During the qualitative phase, participants were interviewed to discover the existing themes in their responses to study questions. Also, in this phase, items were generated on the basis of the extracted themes, and the content validity of the questionnaire was evaluated. The quantitative component included scale development and scale evaluation (Boateng, Neilands, Frongillo, Melgar-Quiñonez & Young, 2018). The study was done in Isfahan, the second metropolis of the country, between May 2017 and March 2021.

2.2. Participants

Our statistical population in the qualitative phase consisted of all married individuals attending 24 municipality family education centers. The initial sample for the qualitative phase consisted of 1065 married individuals (745 women and 320 men), who were randomly selected from those enrolled at a large municipality family education project.

2.3. Phase I - Qualitative phase

2.3.1. Item development

In the first phase, the study's purpose and fundamental concepts of our questions, such as negative reciprocity, positive marital behaviors, and conflict, were verbally explained to participants. Afterward, the study questions were presented verbally, and participants' questions and the ambiguities were answered; then, participants were asked to write their answers on the sheets containing the study questions. The interviews included these open-ended questions: "(a) what is the source of your positive behaviors during your interactions, conflicts, and difficult

moments of the marital relationship? Why do you continue constructive behaviors toward your spouse in such situations? (e.g., humor, empathy, responsiveness, forgiveness, and warmth), (b) When your spouse behaves negatively and destructively, what makes you not reciprocate negatively? (Two quality questions) (c) Despite the conflicts and critical moments in marital life, what are the factors that prevent you from moving toward separation and divorce? (Stability question)."

2.3.2. Item generation

Thematic analysis was used to analyze the data obtained from written interviews. The thematic analysis includes five steps: (a) data familiarization (reading and re-reading of data to get acquainted with all aspects of data prior to starting coding, taking notes about the initial ideas for coding), (b) code generation (coding meaningful and interesting attributes of the data in the whole dataset, collating data related to each code), (c) theme search (analyzing codes and reflection on how several codes may be combined to form a comprehensive theme), (d) themes revision (reading all the coded extracts for each theme and checking whether they constitute a coherent pattern and repeating the same pattern to considering the coherence of themes in relation to the entire data set, generating a thematic map), and (e) theme definition (generating concise and clear definitions and names for themes) (see Braun & Clarke, 2006, for details). The data were collected and analyzed until theoretical saturation was reached.

18 main themes were identified from our interviews. The extracted themes were used to construct scale items. The initial pool of 93 items was developed as much as possible from the participants' direct responses through the inductive method in order to capture the lived experiences of the target population. Then, the themes without all the required items for their presentation were completed by the deductive method (e.g., Resilience and Hardiness themes). The deductive method was conducted on the basis of the existing scales, reviewing the literature and the suggestions from the experts. According to DeVellis (2016) recommendations, efforts were made to write all the items in an understandable, clear, and unambiguous way (DeVellis, 2016).

2.3.3. Content validity

The 93 items pool was sent to four experts in the marital research areas, with a request for their feedback on each item's wording and relevance to the questionnaire's aim. They were also asked to evaluate whether the items covered the essential aspects of themes or not; they could also add comments or suggestions. After implementing the experts' recommendations, the resulting item pool of 135 items was sent to 20 experts in the field for content validity evaluation, and the assessments of 15 of them were received. They rated the items on a 3-point scale - not relevant, slightly relevant, and quite relevant - according to the definition of themes and the research aim. They were also asked to add comments or suggestions. To determine content validity, the content validity index (*CVI*) for items was computed. On the basis of experts' suggestions, 101 items were retained, and some items were changed to be more explicit and readable.

2.4. Phase II –Quantitative phase: scale development and scale evaluation

2.4.1. Pilot study

The first version of the "why level" factors questionnaire, which is called "marital anchors questionnaire" (MAQ) (101 items), was completed by a sample of 40 married individuals (50 %

male, 50 %female) who were randomly selected in Isfahan city to evaluate the clarity of the items. 16 items were removed, and five were modified after this pretesting. The second administration of the new draft of MAQ (85 items) on this sample proved that the items were readable, explicit, and accurate.

2.4.2. Survey administration: Sampling and data collection

Eligible subjects were married individuals in Isfahan city. The sampling strategy was simple random sampling, and the first version of the MAQ (85 items) was administered to 450 married individuals. The number of participants used for validation and factor analysis of this instrument was calculated on the basis of Clark and Watson (2016). Items were rated on a 5-point Likert scale from *1* (strongly disagree) to *5* (strongly agree). The survey was conducted in 14 different regions of Isfahan metropolis. Data were collected by delivering questionnaires to respondents' homes. The completion of questionnaires was anonymous and voluntary. The response rate in survey administration for extraction of factors was 94 %, with a total number of 423 questionnaires returned.

2.5. Validity and reliability of the MAQ

Regarding evaluating the validity, construct validity, convergent, divergent, and known-group validity were examined. Factor analysis was conducted to assess construct validity. Given that the primary aim of the study was to extract the "why level" factors that contribute to a happy and stable marriage, convergent validity was assessed via correlation coefficient with *ENRICH* marital satisfaction scale (Olson, Fournier & Druckman, 1983), and for divergent validity with Marital Instability Index (MII) (Booth, Johnson & Edwards, 1983) in 46 randomly selected married individuals and 96 individuals who were in the final stages of legal dissolution. For known-group validity, differences in MAQ scores between 96 individuals in the final stages of legal dissolution and 96 married individuals were examined. For reliability, reproducibility and internal consistency were examined. For reproducibility, the two-week test-retest reliability was assessed. The same 40 married individuals answered the same questions in the same location two weeks later.

2.6. Data analysis

Construct validity and factor structure were examined by exploratory factor analysis (EFA) using the principal components method with a Varimax rotation. Items with maximum loadings less than 0.30 were excluded from the subsidiary factor analysis. For question 70, factor loadings less than 0.25 were re-runned to fit the appropriate factor. Factor 1 included 34 items. By integrating the qualitative phase findings and the themes extracted in this phase, the factor analysis is performed on the factor 1 items to extract sub-factors relating to the spouse. Three items were removed, one at a time, because of the formation of single-item factors and considering that the theme of these three items was similar to other factors that were extracted.

Convergent and divergent validity of the final version of MAQ (82 items) were evaluated using Pearson's correlation coefficient. For known-group validity, an independent t-test was conducted. Reproducibility was assessed using intraclass correlations. The internal consistency was examined via Cronbach's alpha coefficients. The acceptable level of reliability was considered 0.70 or higher (Cronbach, 1951). Statistical analysis was performed utilizing the Statistical Package for the Social Sciences, version 21.0.

3. Results

3.1. Demographic data

Participants' characteristics are presented in Supplementary Table 1.

3.2. Extracted themes in the qualitative stage

The themes extracted in the qualitative stage were common to the factors extracted in factor analysis, except for four: optimism, spouse's virtues, loving the family unit, and purposefulness. All the themes obtained in response to the two questions of relationship quality were also extracted in response to the relationship stability question.

3.3. Exploratory factor analysis

To assess the factorial validity of the instrument, the 85 items were subjected to EFA. The sampling adequacy measure (Kaiser-Meyer-Olkin) (Kaiser, 1974) was 0.96, and Bartlett's test of sphericity (Bartlett, 1954) was significant (p = 0.00), which suggests that the data were very suitable for analysis. The extracted factors (the primary aim of the study) were: (a) Love, mutual fondness, and mutual understanding, (b) Marital satisfaction, (c) Respect and Positive perception of partner, (d) Low divorce proneness, (e) Resilience and Hardiness, (f) Religion and spirituality, (g) Hope, (h) Children, (i) Negative attitudes toward conflict and divorce and their consequences, (j) Relationship history and long-term investments, (k) Fulfilment of needs, sexual gratification, and perceived support, (l) Virtues: commitment, tendency to forgive, and willingness to sacrifice, (m) Friendship, (n) Financial dependence, and (o) Reputation and stigmatization. Supplementary tables 2 and 3 present MAQ and a summary of exploratory factor analysis results.

3.4. Convergent validity

The correlation between the scores of MAQ and the ENRICH marital satisfaction scale was significant (r (140) = 0.85, p = 0.00).

3.5. Divergent validity

The results showed that the correlation coefficients between the MAQ and Marital Instability Index were significant (r (140) = - 0.90, p = 0.00). According to Cohen (1988), the correlation coefficient obtained for convergent and divergent validity was judged as strong (Cohen, 1988).

3.6. Known-group validity

Independent-samples t-test showed that the difference between the MAQ scores of couples who were in the last stage of legal dissolution (M = 220.67, SD = 55.35) and ordinary married couples (M = 330.47, SD = 43.38) is significant (t *(190)* = 15.29, p = 0 .00).

3.7. Reliability

Cronbach's alpha coefficient of the MAQ was 0.97, indicating high internal consistency. Regarding reproducibility, the two-week test-retest intraclass correlation for the MAQ was 0.70 (p = 0 .00), which was considered a strong agreement (Portney & Watkins, 2009).

References

Bartlett, M. S. (1954). A note on the multiplying factors for various χ2 approximations. *Journal of the Royal Statistical Society: Series B (Methodological), 16*(2), 296-298.

Boateng, G. O., Neilands, T. B., Frongillo, E. A., Melgar-Quiñonez, H. R. & Young, S. L. (2018). Best Practices for Developing and Validating Scales for Health, Social, and Behavioral Research: A Primer. *Front Public Health, 6*149.

Booth, A., Johnson, D. & Edwards, J. N. (1983). Measuring marital instability. *Journal of Marriage and the Family*.

Braun, V. & Clarke, V. (2006). Using thematic analysis in psychology. *Qualitative research in psychology, 3*(2), 77-101.

Cohen, J. (1988). Statistical power analysis for the behavioral sciences, 2nd edn. Á/L, Erbaum Press, Hillsdale, NJ, USA.

Cronbach, L. J. (1951). Coefficient alpha and the internal structure of tests. *psychometrika, 16*(3), 297-334.

DeVellis, R. F. (2016). Scale development: Theory and applications, Sage publications.

Driver, J., Tabares, A., Shapiro, A. F. & Gottman, J. M. (2012). Couple interaction in happy and unhappy marriages: Gottman Laboratory studies.

Kaiser, H. F. (1974). An index of factorial simplicity. *psychometrika, 39*(1), 31-36.

Lewis, R. A. & Spanier, G. B. (1979). Theorizing about the quality and stability of marriage. *Contemporary theories about the family: research-based theories/edited by Wesley R. Burr...[et al.]*.

Olson, D., Fournier, D. & Druckman, J. (1983). PREPARE/ENRICH Counselor's Manual. Minneapolis, MN: PREPARE/ENRICH, Inc.

Portney, L. G. & Watkins, M. P. (2009). Foundations of clinical research: applications to practice, Pearson/Prentice Hall Upper Saddle River, NJ.

Supplementary Table 1.

Demographic information

	**Qualitative Phase (*n* = 1065)**	**Quantitative Phase (*n* = 605)**
Age: Mean (SD)	43.46 (10.73)	38.84 (10.11)
Sex		
Male	320 (30)	264 (43.60)
Female	745 (70)	341 (56.40)
Education		
Illiterate	52 (4.90)	6 (1.00)
Elementary	117 (11)	26 (4.30)
Guidance School to Diploma	552 (51.80)	295 (48.80)
Bachelor's Degree	269(25.30)	229 (37.90)
Master's Degree or Doctorate	75 (7.00)	49 (8.00)
Employment Status		
Employed	164 (15.40)	81 (13.40)
Self-Employed	238 (22.30)	252 (41.70)
Housewife	620 (58.20)	209 (34.50)
Retired	38 (3.60)	24 (4.00)
Unemployed	5 (0.50)	39 (6.40)
Number of children: Mean (SD)	1.87 (1.01)	1.52 (1.18)

Note. All figures are N (%) unless otherwise specified.

Supplementary Table 2.

Marital Anchors Questionnaire (MAQ)

Love, mutual fondness and mutual understanding anchor

1. I love my spouse
2. I love my spouse so much that nothing can separate us
3. My spouse loves me
4. Our love for each other have played a vital role in keeping our marriage stable
5. Because my spouse understands me very well, it is easy for me to endure the hardships of marital life
6. My relationship with my spouse is so close that nothing can disturb it
7. The friendship with mutual understanding has played an important role in maintaining and sustaining our marriage
8. My spouse does her duties well
9. My spouse satisfies my sexual needs well
10. The attraction of my spouse and the fire and passion of seeing her\him makes me forget his\her shortcomings
11. I think my spouse is an ideal person
12. I'm very close to my spouse emotionally and it makes me forgive his\her faults

Marital satisfaction anchor

13. Most of the problems between my spouse and me are minor and transient
14. My spouse has done a lot of good things for me, so his\her shortcomings can be forgiven
15. My spouse has done many good deeds in return for every time he/she has made me upset
16. My spouse has forgiven me many times and it makes me forgive him/her when I am upset
17. The good deeds my spouse has done has played an essential role in maintaining and sustaining our marital life

18. Despite all of the problems that I sometimes have with my spouse, I love our marriage

19. I love my home and my life so much that it can be easy for me to endure the hardships in my relationship with my spouse

20. In spite of all the problems that occur between my spouse and me, living with him\her is sweet

21. I know my destination of life is being with my spouse

22. Feeling completeness with my spouse has played a vital role in maintaining and sustaining our marriage

23. When I compare my marriage with other peoples', I realize that mine is better than many of them

24. I'm really eager for my marital life

Respect and Positive perception of partner anchor

25. Overall, my spouse's good characteristics are more than his/her bad ones

26. When I get very upset with my spouse, subconsciously remembering his/her positive characteristics calms me down

27. The misconducts / wrongdoings of my spouse is intentional and stems from his/her nature

28. Seeing my spouse's positive qualities has played a vital role in maintaining and sustaining our marriage

29. My spouse is a genuine and trustworthy human being

30. My spouse's weaknesses are so numerous that even thinking about it makes me desperate

31. My spouse is worth spending my life with him/her

32. My spouse's humanistic characteristics have played a vital role in maintaining and sustaining our marital life

33. Since no human is perfect, I believe that my spouse's weaknesses are natural

34. Despite the conflicts everyone has with their spouse, I trust and respect my spouse

Low divorce proneness anchor

35. I have no hope of improvement in the conflicts that I have with my spouse

36. Divorce is not so intolerable

37. My spouse makes me more upset than happy
38. It doesn't matter if our marital conflicts are made public
39. In my opinion, we should not accept marital hardships to maintain family unity
40. I do not believe in things like a sense of commitment and responsibility towards a spouse and living together
41. There is no reason to endure the hardships of Marital life
42. I have no longer control over my marriage and relationship with my spouse and I have been discouraged
43. After the divorce there will be better opportunities for marriage or a romantic relationship

Resilience and Hardiness anchor

44. I believe that despite the problems in our marriage, I work to attain my goals
45. When things look hopeless in our relationship, I don't give up
46. I believe that marital problems and hardship make us grow and stronger
47. I can easily deal with hardships and adversities comes in marital life
48. I have overcome marriage adversities so far, therefore I am able to do so in the future
49. I usually tend to bounce back after a conflict or a fight with my spouse
50. When I am faced with a new problem in our marital life I don't fall apart
51. I do not give up easily when good marital conditions collapse
52. My endurance and hardiness during the difficulties and hardships of marriage, plays a vital role in maintaining and sustaining our relationship

Religion and spirituality anchor

53. Seeking approval from God has sustained my marital life
54. Trusting in God makes it easy for me to endure the hardships of marital life

55. Belief in God and His commands have played a vital role in maintaining and sustaining our marital life

Hope anchor

56. I always hope for a positive change in my spouse

57. When I quarrel with my spouse, hope for the future calms me down

58. Hope for a better marital relationship has played a vital role in maintaining and sustaining our marriage

Children anchor

59. Having children causes one to cope with the hardships of a marriage

60. Love towards my children makes me avoid a serious fight or quarrel with my spouse

61. Having children plays a vital role in maintaining and sustaining the marriage

Negative attitudes toward conflict and divorce and their consequences anchor

62. I prefer not to think about divorce because divorce has worrying consequences

63. I hate divorce and separation

64. My awareness of the consequences of marital conflicts or divorce has played a vital role in maintaining and sustaining our marital life

Relationship history and long-term investments anchor

65. I have not built up my marriage easily, so I do not want to lose it easily

66. My spouse and I have been through so much, and this makes me forgive him/her when we have a conflict

67. The long-term investments in our relationship has a crucial role in maintaining and sustaining our marriage

Fulfilment of needs, sexual gratification, and perceived support anchor

68. Without my spouse, it becomes challenging to satisfy my sexual, physical, and psychological needs

69. The needs that I have for my spouse have played a vital role in maintaining and sustaining our marital life

70. I have no better support than my spouse

Virtues: commitment, tendency to forgive, and willingness to sacrifice anchor

71. When you marry someone, you must stand until the end

72. When I have a conflict with my spouse, my belief in humanistic values such as forgiveness calms me down

73. My commitment to humanistic values such as sacrifice has played a vital role in maintaining and sustaining our marital life

Friendship anchor

74. Because I don't like my spouse, her shortcomings have become unbearable

75. Our relationship is Like a relationship of two close friends

76. I have never felt that I'm friends with my spouse

Financial dependence anchor

77. Without my spouse I can also have financial independence

78. If necessary, I won't need my spouse's income

79. If necessary, I have an independent income from my spouse

Reputation and stigmatization anchor

80. In my opinion, conflict with my spouse or separation has a very negative effect on our reputation

81. If our relationship or marriage was in trouble, I would try not to let relatives and friends know about it

82. The reputation we have among relatives and friends has played a vital role in maintaining and sustaining our marriage

Supplementary Table 3.

Summary of Exploratory Factor Analysis Results for Marital Anchors Questionnaire Using Principal Components Method with Varimax Rotation (n = 423).

	**Factor loadings**														
Item	1	2	3	4	5	6	7	8	9	10	11	12	13	14	15
Factor 1: A) Love, mutual fondness and mutual understanding															
1.	**.64**														
2.	**.64**														
3.	**.54**														
4.	**.65**														
5.	**.75**														
6.	**.72**														
7.	**.68**														
8.	**.57**														
9.	**.41**														
10.	**.50**														
11.	**.65**														
12.	**.58**														
Factor 1: B) Marital satisfaction															
13.		**.47**													

14.	**.52**			
15.	**.53**			
16.	**.49**			
17.	**.40**			
18.	**.69**			.40
19.	**.73**			.34
20.	**.69**			
21.	**.57**			
22.	**.63**			
23.	**.56**			
24.	**.50**			
Factor 1: C) Respect and Positive perception of partner				
25.		**.50**		
26.		**.33**		
27.		**.75**	.34	
28.		**.46**		
29.		**.47**		
30.		**.74**	.39	
31.		**.44**		
32.		**.49**		

33.		**.35**		.34					
34.		**.31**		.30					
Factor 2: Low divorce proneness									
35.	.41		**.57**						
36.			**.61**						
37.			**.44**						
38.			**.65**						
39.			**.66**						
40.			**.61**						
41.			**.64**						
42.	.40		**.54**						
43.			**.48**						
Factor 3: Resilience and Hardiness									
44.				**.51**					
45.				**.51**					
46.				**.46**					
47.	.40			**.58**					
48.				**.51**					.35
49.				**.41**					
50.				**.54**					
51.				**.69**					

52.				**.38**					
Factor 4: Religion and spirituality									
53.					**.85**				
54.					**.83**				
55.					**.85**				
Factor 5: Hope									
56.		.35				**.62**			
57.	38					**.46**			
58.	.42					**.59**			
Factor 6: Children									
59.							**.78**		
60.							**.75**		
61.							**.67**		
Factor 7: Negative attitudes toward conflict and divorce and their consequences									
62.								**.34**	
63.								**.69**	
64.								**.44**	
Factor 8: Relationship history and long-term investments									
65.									**.44**

66.	.44							**.60**				
67.					.36			**.38**				
Factor 9: Fulfilment of needs, sexual gratification, and perceived support												
68.		.38							**.63**			
69.		.38							**.54**			
70.		.61							**.26**			
Factor 10: Virtues: commitment, tendency to forgive, and willingness to sacrifice												
71.										**.63**		
72.			.31							**.48**		
73.										**.49**		
Factor 11: Friendship												
74.											**.50**	
75.											**.34**	
76.	.32										**.69**	
Factor 12: Financial dependence												
77.												**.77**
78.												**.87**
79.												**.89**

Factor 13: Reputation and stigmatization															
80.										.35					**. 42**
81.															**.30**
82.															**.68**
Eigenvalues		26.513		4.22	3.01	2.21	1.86	1.58	1.52	1.41	1.32	1.23	1.23	1.16	1.12
	17.18	1.30	1.15												
% of variance		32.33		5.15	3.67	2.69	2.27	1.92	1.85	1.72	1.61	1.50	1.49	1.41	1.37
	50.54	3.81	3.38												

Note: Reverse-scored items: 27, 30, 35, 36, 37, 38, 39, 40, 41, 42, 43, 75, 77, 78, 79, 80

FIGURE 1.

The study flow diagram.

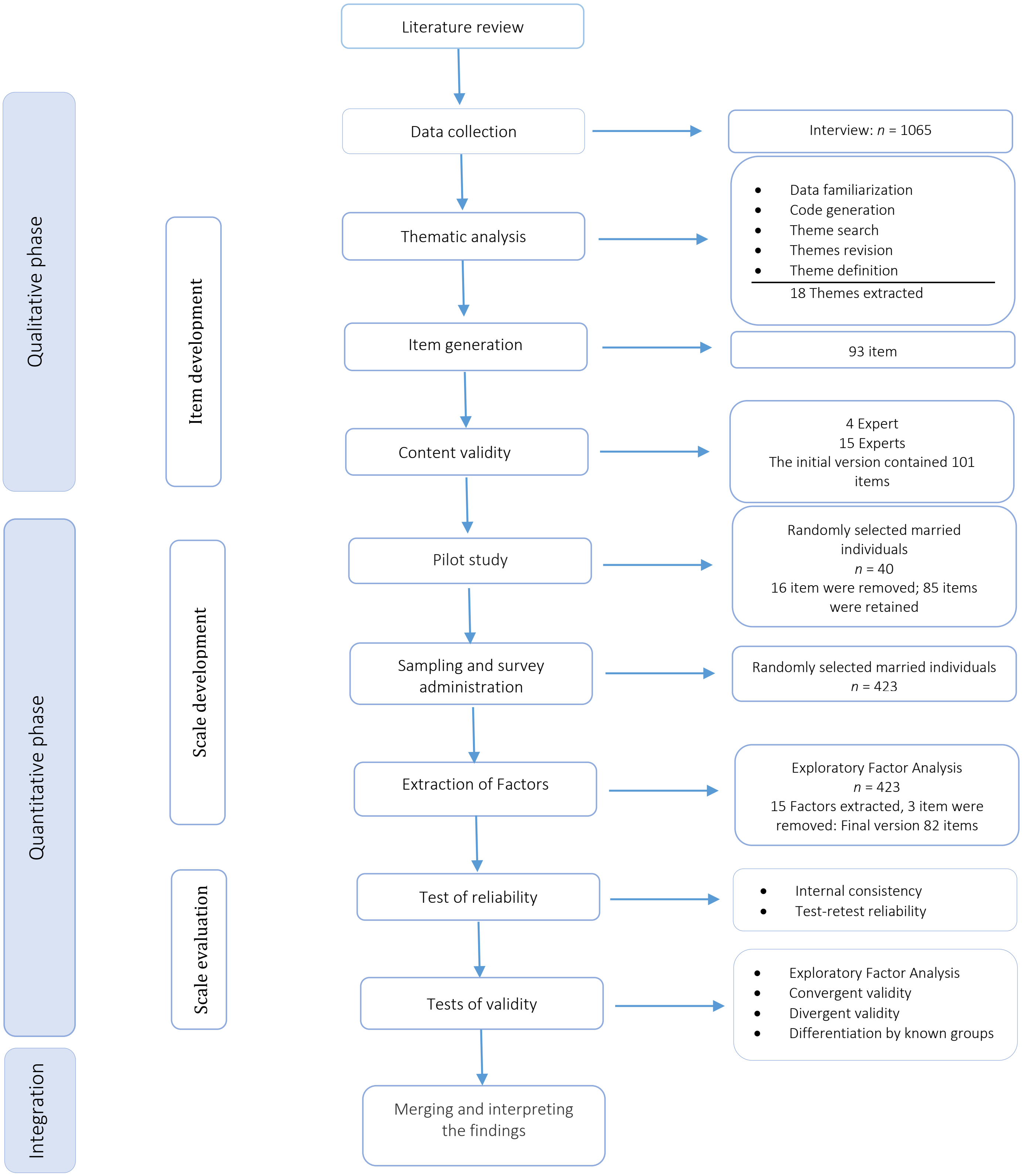

Supplement: Supplementary file 1 [file Data_Sheet_1.pdf]
